# IGANets: Isogeometric analysis networks and their applications to linear structural analysis problems

**DOI:** 10.1007/s00366-026-02312-6

**Published:** 2026-05-11

**Authors:** Matthias Möller, Günther Obermair, Isabella Singer, Christian Gollmann, Alessandro Reali, Stefanie Elgeti

**Affiliations:** 1 Department of Applied Mathematics, Delft University of Technology, Mekelweg 4, 2628 CD Delft, The Netherlands; 2 Institute of Lightweight Design and Structural Biomechanics, TU Wien, Gumpendorferstr. 7, 1070 Vienna, Austria; 3 Department of Civil Engineering and Architecture, University of Pavia, via A.Ferrata 3, 27100 Pavia, Italy

**Keywords:** Physics-informed machine learning, Surrogate models, Real-time design, Isogeometric collocation

## Abstract

Fast numerical predictions have become an indispensable component of modern engineering design workflows, whether in interactive design within computer-aided design (CAD) environments or in multi-query numerical tasks such as design optimization and uncertainty quantification. Depending on the context, “fast” may refer to near real-time predictions within a few seconds, or simply to methods that are significantly faster than high-fidelity simulations, for example those based on the finite element method (FEM). With the aim of providing a tool that not only enables such accelerated predictions but also integrates seamlessly into established workflows, we introduce the concept of IGANets. IGANets are spline-based, physics-informed machine learning models that can be integrated naturally between CAD representations and numerical analysis tools, particularly those based on isogeometric analysis (IGA). Unlike purely data-driven approaches, IGANets do not inherently rely on precomputed training data; instead, they are formulated in a collocation setting directly from physical models. In this paper, we present the IGANets concept and demonstrate its feasibility through numerical experiments for the Poisson equation and linear elasticity. In addition, we investigate a multi-instance linear-elasticity setting with varying I-beam-like geometries and boundary conditions in order to assess the generalization capability of the framework. The results show that IGANets can predict solutions for previously unseen problem instances within the training range with improved accuracy as the number of training samples increases.

## Introduction

In modern engineering design workflows, numerical analysis plays a crucial role in providing insight into the performance of engineering components even before fabrication. Conceptually, this can be described as the creation of a digital twin prototype – a virtual representation of the physical component that allows for comprehensive investigation, evaluation, and optimization prior to manufacturing. Conventional numerical methods, such as finite element analysis, are often computationally intensive and time-consuming, which prevents their integration into real-time, interactive design processes. By "real-time," we refer to timescales compatible with the designer’s workflow, typically in the order of seconds or less per interaction.

Within this context, this paper introduces IGANet, a novel methodology aimed at enabling real-time digital twin prototyping with particular emphasis on geometry-driven design and varying loading or usage scenarios. The proposed approach empowers designers to obtain immediate feedback on the physical behavior of design artifacts as they modify geometry or boundary conditions, thereby fostering an interactive and informed design process. IGANets combine the mathematical framework of isogeometric analysis (IGA) with the concept of physics-informed neural operators. Parametric objects (e.g., geometries, boundary conditions, materials) are encoded through their coefficients relative to fixed spline bases and provided as inputs to a neural network. The network outputs the solution to parametric partial differential equations (PPDE) in the form of spline coefficients. Building on the theory of isogeometric collocation methods, IGANets yield converged solutions upon increasing the number of unknowns alongside the sampling points. The physics model is implemented in the loss function in a least-squares sense, thereby leveraging the higher continuity of splines to represent higher derivatives without the need to introduce auxiliary variables. IGANets are trained over a family of inputs that represent relevant instances of the expected design space. The training can be performed in full self-supervised manner or assisted by available simulation/experimental data, thereby overcoming the major roadblocks of existing methods. The ultimate goal of this work is to bridge the gap between high-fidelity physical simulation and real-time interactivity, thereby facilitating both manual and automated optimization processes within digital design environments. The comparison of IGANets with the state of the art is discussed in the following.

A digital twin prototype (DTP) is a virtual representation or simulation of a physical object, system, or process enabled through digital technologies, i.e., computer-aided design (CAD) and analysis (CAA). One way in which a DTP can be obtained are surrogate modelling techniques. The generation of such surrogate models is based on the fast generation of accurate and reliable solutions of parametric partial differential equations (PPDE), where the considered parameters can be geometric shapes, material parameters, etc. Surrogate modelling approaches suitable for design processes can be grouped as follows ( [1]): (1) dimensional/analytical model reduction (e.g., 3D-to-2D or 2.5D reduction by exploiting symmetries, replacement of nonlinear by linear model equations); (2) computational/model-order reduction (e.g., reduced basis (RB) method or proper orthogonal decomposition (POD)); (3) physics-informed machine learning (e.g., physics-informed neural networks (PINN), deep operator networks (DeepONets)).

IGANets fall into the last category, so in the following, we will briefly describe seminal technologies in the field of physics-informed machine learning and then give an overview over contributions within the frame of linear elasticity. Within this domain, one method stands out as a particularly natural fusion of machine learning and scientific computing – physics-informed neural networks (PINNs). Although the foundational ideas can be traced back to early work in the 1990s [2–4], PINNs only gained widespread attention and rapid development following their reinvention by Raissi, Perdikaris, and Karniadakis in 2017 [5]. The core concept of PINNs is to approximate the solution of physical problems governed by (partial) differential equations. In contrast to conventional supervised learning approaches that rely solely on input-output data pairs, PINNs incorporate the governing physical laws directly into the learning process by embedding the differential equations into the loss function of the neural network. This integration enables the model to learn physically consistent solutions, even in the absence of extensive labeled data. In the context of linear elasticity, PINNs have for example been employed in the following works [6–12].

Instead, deep operator networks (DeepONets) are a class of neural network architectures designed to learn nonlinear operators – that is, mappings between infinite-dimensional function spaces. Unlike PINNs that approximate functions, DeepONets aim to approximate operators, such as the solution map of a differential equation. Introduced by Lu et al. [13, 14], a DeepONet consists of two subnetworks: a branch network, which processes input functions (typically represented by pointwise evaluations), and a trunk network, which handles the evaluation locations. Given their superior generalization capabilities as compared to PINNs, DeepONets are particularly well-suited for solving families of parametric PDEs. In the field of linear elasticity, DeepONets have for example been applied in the following works [15, 16].

In addition to these two main variants of physics-informed machine learning, a wide range of alternative approaches has emerged, offering diverse strategies for integrating physical knowledge into learning frameworks. A comprehensive review of these methods can be found in [17].

Another central concept in IGANets are collocation approaches. Collocation methods solve PDEs by enforcing the governing equations at a discrete set of points, known as collocation points [18]. These methods originated in the 1960s and 70s as a way to solve boundary value problems for ordinary and partial differential equations [19–21], offering a simpler alternative to weighted residual or Galerkin methods by avoiding domain integration. A modern and efficient extension is spline-based collocation [22–26], which integrates collocation with the spline-based geometry representation used in CAD; thereby exploiting the smoothness and geometric exactness of spline basis functions. A further development is least-squares collocation [27–29], where the differential equation is not enforced exactly at each collocation point but rather in a least-squares sense by minimizing the squared residuals over all points. This relaxation improves robustness, particularly when dealing with noisy data, over-constrained systems, or ill-posed problems.

In the remainder of the paper, the IGANets approach is introduced in Section 2, followed by a concept validation based on the Poisson equation in Section 3. The application of IGANets to linear elasticity is demonstrated in Section 4.

## The IGANets approach

To introduce the IGANets approach, we consider the abstract boundary-value problem (BVP)

1 $$\begin{aligned} {\textbf{x}}\in \Omega \,: \quad {\mathcal {R}}_\Omega [u; f, \boldsymbol{\alpha }]({\textbf{x}})&= 0, \end{aligned}$$

2 $$\begin{aligned} {\textbf{x}}\in \Gamma \,: \quad {\mathcal {R}}_\Gamma [u; g, \boldsymbol{\alpha }]({\textbf{x}})&= 0, \end{aligned}$$

where ${\mathcal {R}}_\Omega$ and ${\mathcal {R}}_\Gamma$ are the differential operators in residual form acting on the solution *u* in the domain $\Omega$ and its boundary $\Gamma =\partial \Omega$, respectively. Here, *f* and *g* denote possible right-hand sides and boundary values, and $\boldsymbol{\alpha }$ represents problem-specific parameters such as the Lamé parameters utilized in Section 4.1.3.

Simply put, the task of any numerical method for solving BVPs is to find a mapping

3 $$\begin{aligned} {\mathcal {M}}\,:\, \left( \Omega ,\Gamma ,f,g,\boldsymbol{\alpha }\right) \mapsto u \end{aligned}$$

such that Equations (1)-(2) are satisfied for all ${\textbf{x}}$ in the domain and its boundary.

Although traditional physics-informed machine learning approaches often hardcode the geometry, right-hand sides, and boundary values into the loss function and learn over a range of problem parameters $\boldsymbol{\alpha }$, the core idea of IGANets is to encode the entire problem set-up in terms of basis coefficients relative to a priori chosen bases. The basis coefficients are then fed into the neural network as inputs and the network outputs are reinterpreted as coefficients of the solution relative to an again a priori determined basis.

This is in stark contrast to classical PINNs, where the network input is a physical coordinate in the domain or at its boundary and the network’s task is to predict the point-wise solution value at that location. Although this concept ensures a very small number of network inputs and outputs, it makes it nontrivial to provide complex problem set-ups (i.e., $\Omega , \Gamma , f, g$) as network input so that even parameterized PINNs are often restricted to learn over only a few problem parameters $\boldsymbol{\alpha }$ that can be provided as scalar inputs.

The IGANets approach also differs from the concept of DeepONets, where the basis is not determined a priori but learned in a separate network (termed *branch network*). Although this concept is particularly interesting when the ‘optimal’ basis is not known a priori, e.g., when the solution has sharp localized features such as shock fronts, the IGANets approach is designed as a learning-based drop-in replacement for classical isogeometric analysis (IGA) and, as such, it builds on the idea of representing both the geometry and the solution in compatible spline spaces.

For simplicity, we illustrated the IGANets concept in two dimensions and for a scalar BVP but remark that it naturally generalizes to higher dimensions, vector-valued problems, and even non-tensor-product spline spaces. To begin with, let

4 $$\begin{aligned} {\mathcal {S}}=S^{p,c}(\Xi )\otimes S^{p,c}(\Xi ) \end{aligned}$$

denote the space of bivariate B-spline basis functions $b_ib_j\in {\mathcal {S}}$, each of degree *p* and continuity $c\le p-1$, defined on the open knot vector

5 $$\begin{aligned} \Xi =[\underbrace{\xi _1=\xi _2=\dots =\xi _{p+1}}_{p+1 \text { times}}, \dots , \xi _i, \dots , \underbrace{\xi _{n}=\dots = \xi _{n+p+1}}_{p+1 \text { times}}] \end{aligned}$$

consisting of $n+p+1$ knots – a sequence of non-decreasing real values from the interval [0, 1] – and, hence, *n* basis functions per dimension, respectively.

The exact geometry $\Omega \subset {\mathbb {R}}^2$ can then be discretized as

6 $$\begin{aligned} \boldsymbol{\xi }\in {{\hat{\Omega }}} \,:\quad {\hat{\textbf{x}}}_h(\boldsymbol{\xi })=\sum _{i=1}^{n}\sum _{j=1}^{n} b_i(\xi ^{(1)}) b_j(\xi ^{(2)}) {\textbf{x}}_{ij}, \quad {\textbf{x}}_{ij}\in {\mathbb {R}}^2, \end{aligned}$$

which maps from the parametric domain ${{\hat{\Omega }}} = [0,1]^2$ to the physical domain $\Omega _h$ and its boundary $\Gamma _h$. If the forward mapping defined in (6) is bijective, its inverse, the so-called pull-back mapping, is well defined

7 $$\begin{aligned} {\textbf{x}}\in \Omega _h\,:\quad \boldsymbol{\xi }_h({\textbf{x}})={\hat{\textbf{x}}}_h^{-1}({\textbf{x}}). \end{aligned}$$

The solution *u*, the right-hand side *f* and the boundary values *g* are approximated in the same spline space

8 $$\begin{aligned} {\textbf{x}} \in \Omega _h \,:\quad u_h({\textbf{x}})&=\sum _{i=1}^{n}\sum _{j=1}^{n} b_i(\xi _{h}^{(1)}({\textbf{x}})) b_j(\xi _{h}^{(2)}({\textbf{x}})) u_{ij}, \quad&u_{ij}\in {\mathbb {R}}, \end{aligned}$$

9 $$\begin{aligned} {\textbf{x}} \in \Omega _h \,:\quad f_h({\textbf{x}})&=\sum _{i=1}^{n}\sum _{j=1}^{n} b_i(\xi _{h}^{(1)}({\textbf{x}})) b_j(\xi _{h}^{(2)}({\textbf{x}})) f_{ij}, \quad&f_{ij}\in {\mathbb {R}}, \end{aligned}$$

10 $$\begin{aligned} {\textbf{x}} \in \Gamma _h \,:\quad g_h^\gamma ({\textbf{x}})&=\sum _{i=1}^{n} b_i(\xi _{h}^{d(\gamma )}({\textbf{x}})) g_{i}, \quad&g_{i}\in {\mathbb {R}}, \end{aligned}$$

where $\xi _{h}^{(d)}$ denotes the *d*th parametric direction of the inverse map defined in (7), $\gamma \in \Gamma _h$ is a boundary segment, and $d(\gamma )$ is defined to select the spatial direction that corresponds to the boundary segment $\gamma$. As an example, a segment $\gamma$ located at the upper or lower boundary of the unit square would be mapped to the first parametric direction $\xi _{h}^{(1)}$, whereas segments at the left and right boundary would be mapped to $\xi _{h}^{(2)}$. Let us remark that in principle each of the above quantities can be defined in a different, not necessarily tensor-product, spline space.

With the above definitions, the task of the IGANets (and a puristic IGA solver) is to find a mapping

11 $$\begin{aligned} {\mathcal {M}}_h \,:\, \left( \left\{ {\textbf{x}}_{ij}\right\} , \left\{ f_{ij}\right\} , \left\{ g_{i}\right\} , \boldsymbol{\alpha } \right) \mapsto \left\{ u_{ij}\right\} \end{aligned}$$

such that the discretized counterparts of Equations (1)-(2) are satisfied for a finite set of sampling points ${\textbf{x}}_k$ in the domain and its boundary. This can be accomplished by concatenating all flattened^1^ inputs to a single vector^2^

12 $$\begin{aligned} \textit{input}:=\text {concat}\left( \text {flat}\left\{ {\textbf{x}}_{ij}\right\} , \text {flat}\left\{ f_{ij}\right\} , \text {flat}\left\{ g_{i}\right\} , \text {flat}\left( \boldsymbol{\alpha }\right) \right) \in {\mathbb {R}}^{3n^2+4n+m} \end{aligned}$$

with *m* being the number of parameters $\boldsymbol{\alpha }$ and feeding it as input into a feed-forward neural network. The network’s flat output is then reshaped^3^ into an $n\times n$ matrix whose entries are the solution coefficients relative to the specified B-spline basis:

13 $$\begin{aligned} \left\{ u_{ij}\right\} :=\text {reshape}\left( \textit{output}, n, n\right) \in {\mathbb {R}}^{n^2} \end{aligned}$$

The network’s loss function is defined as

14 $$\begin{aligned} \text {loss} = \text {loss}_\text {pde} + \text {loss}_\text {bdr}. \end{aligned}$$

The previous (14) is comprised of the two components

15 $$\begin{aligned} \text {loss}_\text {pde}&= \frac{\omega _\Omega }{N_\Omega } \sum _{k=1}^{N_\Omega } \left( {\mathcal {R}}_{\Omega _h}[u_h; f_h, \boldsymbol{\alpha }]({\textbf{x}}_k)\right) ^2, \end{aligned}$$

16 $$\begin{aligned} \text {loss}_\text {bdr}&= \frac{\omega _\Gamma }{N_\Gamma } \sum _{k=1}^{N_\Gamma } \left( {\mathcal {R}}_{\Gamma _h}[u_h; f_h, \boldsymbol{\alpha }]({\textbf{x}}_k)\right) ^2, \end{aligned}$$

where $N_\Omega$ and $N_\Gamma$ are the number of sampling points ${\textbf{x}}_k$ in the domain and on the boundary, respectively, and $\omega _\Omega$ and $\omega _\Gamma$ are weights to balance between the two components of the loss function. Inspired by the isogeometric collocation method by [23], we choose the sampling points as the images of the *Greville abscissae*

17 $$\begin{aligned} {\bar{\xi }}_k = \frac{\xi _{k+1}+\cdots +\xi _{k+p}}{p} \end{aligned}$$

under the push-forward mapping, i.e., ${\textbf{x}}_k={\hat{\textbf{x}}}_h(\boldsymbol{{\bar{\xi }}}_k)$, or that of a virtually refined spline space leading to a learning-based counterpart of the isogeometric least-squares collocation method by [29]. In both cases, some parts of the loss function simplify, e.g., the basis functions in Equations (9)-(10) can be evaluated at $\boldsymbol{{\bar{\xi }}}_k$ directly.

As an alternative to the weak imposition of boundary conditions through residual terms in the loss function, Dirichlet boundary conditions can be imposed strongly by either overwriting the corresponding entries of the network output by the coefficients of the Dirichlet values and excluding the corresponding terms in the loss functions or by eliminating the solution coefficients corresponding to Dirichlet values from the network output and augmenting the reduced output by the prescribed coefficients afterwards. Both approaches overcome the general challenge of physics-enhanced neural networks to enforce Dirichlet boundary conditions accurately through the careful tuning of the weighting coefficient $\omega _\Gamma$, as for example seen for PINNs in [30]. The latter approach moreover leads to slightly smaller networks but requires some bookkeeping in the reinterpretation of the network output as spline function, cf. (13).

It is furthermore possible to augment this purely physics-informed loss function by a supervised data component, e.g.,

18 $$\begin{aligned} \text {loss}_\text {data} = \frac{\omega _\text {data}}{N_\text {data}} \sum _{k=1}^{N_\text {data}} \left( u_h({\textbf{x}}_k)-u_\text {data}({\textbf{x}}_k)\right) ^2. \end{aligned}$$

**Fig. 1:**
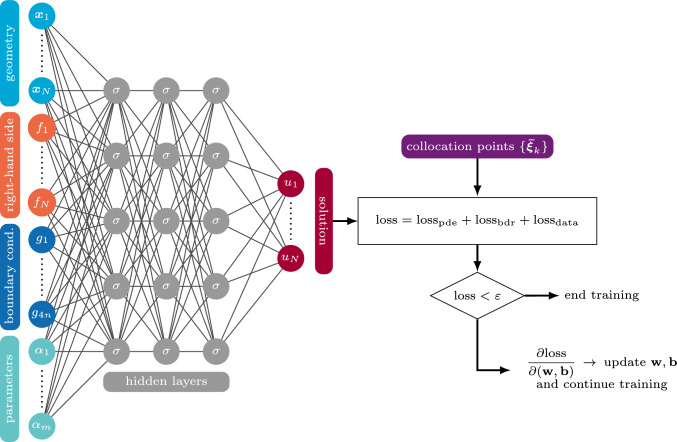
General working principle of IGANets

The overall working principle of IGANets is depicted in Fig. 1, whereby $N=n^2$.

## Concept validation for the Poisson problem

In this section, we demonstrate the correct functioning of the proposed IGANets concept for the two-dimensional Poisson problem solved on the unit square $\Omega =[0,1]^2$ with homogeneous Dirichlet boundary conditions, i.e.,

19 $$\begin{aligned} \Delta u&= f \, \quad \text {in } \Omega ,\end{aligned}$$

20 $$\begin{aligned} u&= 0 \, \quad \text {on } \Gamma . \end{aligned}$$

The right-hand side is set to

21 $$\begin{aligned} f(x,y)=-2\pi ^2\sin (\pi x)\sin (\pi y) \end{aligned}$$

so that the exact solution equals

22 $$\begin{aligned} u(x,y)=\sin (\pi x)\sin (\pi y). \end{aligned}$$

Let *u* and *f* be approximated in the spline space $S^{p,p-1}(\Xi )\otimes S^{p,p-1}(\Xi )$, where $\Xi$ is an open uniform knot vector consisting of *n* equidistant knots. The coefficients $f_{ij}$ in (9) are obtained via B-spline interpolation at the Greville abscissae $\{\boldsymbol{{\bar{\xi }}}_k\}_k$.

Putting it all together, the IGANets’ loss function reads

23 $$\begin{aligned} \text {loss} = \frac{\omega _\Omega }{N_\Omega } \sum _{k=1}^{N_\Omega } \left( \Delta u_h(\boldsymbol{\bar{\xi }}_k) - f_h(\boldsymbol{\bar{\xi }}_k)\right) ^2 + \frac{\omega _\Gamma }{N_\Gamma } \sum _{k=1}^{N_\Gamma } \left( u_h(\boldsymbol{\bar{\xi }}_k) \right) ^2, \end{aligned}$$

where the homogeneous Dirichlet values simplify the boundary loss term. Since IGANets adopt the classical FEM/IGA paradigm for computing derivatives of their outputs with respect to physical variables, derivative evaluation reduces to the appropriate differentiation of the basis functions, e.g.,

24 $$\begin{aligned} \Delta u_h(\boldsymbol{\xi }) = \sum _{i=1}^{n}\sum _{j=1}^{n} \left( b_i^{\prime \prime }(\xi ^{(1)}) b_j(\xi ^{(2)}) + b_i(\xi ^{(1)}) b_j^{\prime \prime }(\xi ^{(2)}) \right) u_{ij}. \end{aligned}$$

It is worth noting that this stands in contrast to PINNs, DeepONets, and other physics-informed machine learning approaches for differential equations that compute derivatives of the network outputs with the help of algorithmic differentiation (AD). The involved computational costs and memory consumption especially for higher-order derivatives are considered one of the major limitations of AD-based approaches, whereas the computational costs and memory consumption for computing spatial derivatives in IGANets are comparable to that of classical FEM/IGA formulations.

Consequently, computationally expensive and memory consuming algorithmic differentiation is only used for computing the derivatives of the loss function with respect to the network weights. As such, the computational costs are independent of the order of the differential equations, which is not the case in other physics-informed machine learning approaches. For an efficient algorithm to evaluate multi-variate B-spline basis functions and their derivatives, the reader is referred to Appendix A.

**Table 1:** Comparison between IGANets and the collocation IGA method for bi-cubic ($p=3$) tensor-product B-splines

#coeffs	#weights	#epochs	MSE_loss_	MSE_IGANets_	#iter	MSE_C-IGA_
1 layer, 1 neuron, Sigmoid activation function, $\omega _\Omega =1$, $\omega _\Gamma =1$						
4 × 4	73	1	1.37 · 10^+1^	3.36 · 10^−2^	1	1.31 · 10^−3^
8 × 8	233	6	1.37 · 10^−3^	1.46 · 10^−4^	86	9.86 · 10^−5^
16 × 16	841	65	3.19 · 10^−6^	**3.89** ·** 10**^**−6**^	297	**3.88** ·** 10**^**−6**^
32 × 32	3209	930	3.85 · 10^−6^	**3.59** ·** 10**^**−7**^	1408	**1.96** ·** 10**^**−7**^
64 × 64	12553	2340	6.14 · 10^−5^	1.49 · 10^−5^	> 10000	1.11 · 10^−8^
1 layer, 1 neuron, Sigmoid activation function, $\omega _\Omega =1$, $\omega _\Gamma =10^3$						
4 × 4	73	1	1.87 · 10^+1^	1.09 · 10^−2^	1	1.31 · 10^−3^
8 × 8	233	1	1.44 · 10^−3^	**9.79** ·** 10**^**−5**^	86	**9.86** ·** 10**^**−5**^
16 × 16	841	24	3.07 · 10^−6^	**3.88** ·** 10**^**−6**^	297	**3.88** ·** 10**^**−6**^
32 × 32	3209	65	1.79 · 10^−8^	**1.97** ·** 10**^**−7**^	1408	**1.96** ·** 10**^**−7**^
64 × 64	12553	315	3.19 · 10^−8^	**1.26** ·** 10**^**−8**^	> 10000	**1.11** ·** 10**^**−8**^
10 layers, 10 neurons per layer, Sigmoid activation function, $\omega _\Omega =1$, $\omega _\Gamma =1$						
4 × 4	1576	3	1.37 · 10^+1^	3.36 · 10^−2^	1	1.31 · 10^−3^
8 × 8	2744	16	1.37 · 10^−3^	1.46 · 10^−4^	86	9.86 · 10^−5^
16 × 16	7096	467	3.08 · 10^−6^	**3.89** ·** 10**^**−6**^	297	**3.88** ·** 10**^**−6**^
32 × 32	23864	1225	1.76 · 10^−7^	**1.27** ·** 10**^**−7**^	1408	**1.96** ·** 10**^**−7**^
64 × 64	89656	4557	2.53 · 10^−6^	8.09 · 10^−7^	> 10000	1.11 · 10^−8^
10 layers, 10 neurons per layer, Sigmoid activation function, $\omega _\Omega =1$, $\omega _\Gamma =10^3$						
4 × 4	1576	2	1.71 · 10^+1^	1.09 · 10^−2^	1	1.31 · 10^−3^
8 × 8	2744	4	1.44 · 10^−3^	**9.79** ·** 10**^**−5**^	86	**9.86** ·** 10**^**−5**^
16 × 16	7096	131	3.07 · 10^−6^	**3.88** ·** 10**^**−6**^	297	**3.88** ·** 10**^**−6**^
32 × 32	23864	285	1.92 · 10^−8^	**1.95** ·** 10**^**−7**^	1408	**1.96** ·** 10**^**−7**^
64 × 64	89656	575	1.91 · 10^−8^	**1.09** ·** 10**^**−8**^	> 10000	**1.11** ·** 10**^**−8**^

Values where both methods yield results with comparable accuracy are marked in bold. From left to right, the columns indicate the number of coefficients/basis functions (#coeffs), the number of network weights (#weights), the number of epochs until loss-value stagnation (#epochs), the mean-squared loss value ($\text {MSE}_\text {loss}$), the mean-squared error between the IGANets’ solution and the exact solution interpolated in the spline space ($\text {MSE}_\text {IGANets}$), the number of BiCGStab iterations of the collocation IGA method (#iter), and the mean-squared error between the collocation IGA solution and the exact solution interpolated in the spline space ($\text {MSE}_\text {C-IGA}$)

**Table 2:** Comparison between IGANets and the collocation IGA method for bi-quartic ($p=4$) tensor-product B-splines

#coeffs	#weights	#epochs	MSE_loss_	MSE_IGANets_	#iter	MSE_C-IGA_
1 layer, 1 neuron, Sigmoid activation function, $\omega _\Omega =1$, $\omega _\Gamma =1$						
5 × 5	104	4	3.29 · 10^−2^	1.37 · 10^−4^	12	4.47 · 10^−5^
10 × 10	349	17	2.78 · 10^−5^	8.90 · 10^−7^	48	2.19 · 10^−9^
20 × 20	1289	99	1.19 · 10^−7^	3.23 · 10^−9^	582	3.24 · 10^−12^
40 × 40	4969	1530	5.11 · 10^−6^	7.53 · 10^−7^	1511	7.71 · 10^−15^
80 × 80	19529	> 5000	2.75 · 10^−3^	2.17 · 10^−4^	> 10000	7.69 · 10^−11^
1 layer, 1 neuron, Sigmoid activation function, $\omega _\Omega =1$, $\omega _\Gamma =10^3$						
5 × 5	104	5	3.29 · 10^−2^	1.37 · 10^−4^	10	4.47 · 10^−5^
10 × 10	349	6	2.60 · 10^−5^	**1.84** ·** 10**^**−9**^	48	**2.19** ·** 10**^**−9**^
20 × 20	1289	2164	4.36 · 10^−8^	**3.04** ·** 10**^**−12**^	582	**3.24** ·** 10**^**−12**^
40 × 40	4969	> 5000	5.33 · 10^−9^	3.54 · 10^−12^	1511	7.71 · 10^−15^
80 × 80	19529	> 5000	7.57 · 10^−8^	3.14 · 10^−11^	> 10000	7.69 · 10^−11^
10 layers, 10 neurons per layer, Sigmoid activation function, $\omega _\Omega =1$, $\omega _\Gamma =1$						
5 × 5	1805	4	3.29 · 10^−2^	1.37 · 10^−4^	12	4.47 · 10^−5^
10 × 10	3580	6	2.58 · 10^−5^	5.40 · 10^−8^	48	2.19 · 10^−9^
20 × 20	10280	> 5000	3.11 · 10^−7^	4.40 · 10^−8^	582	3.24 · 10^−12^
40 × 40	36280	> 5000	1.09 · 10^−6^	2.34 · 10^−7^	1511	7.71 · 10^−15^
80 × 80	138680	> 5000	5.34 · 10^−4^	8.67 · 10^−5^	> 10000	7.69 · 10^−11^
10 layers, 10 neurons per layer, Sigmoid activation function, $\omega _\Omega =1$, $\omega _\Gamma =10^3$						
5 × 5	1805	3	3.29 · 10^−2^	1.37 · 10^−4^	12	4.47 · 10^−5^
10 × 10	3580	17	2.60 · 10^−5^	**1.84** ·** 10**^**−9**^	48	**2.19** ·** 10**^**−9**^
20 × 20	10280	1206	4.37 · 10^−8^	**3.26** ·** 10**^**−12**^	582	**3.24** ·** 10**^**−12**^
40 × 40	36280	318	2.92 · 10^−8^	5.49 · 10^−11^	1511	7.71 · 10^−15^
80 × 80	138680	1629	8.12 · 10^−7^	1.91 · 10^−9^	> 10000	7.69 · 10^−11^

Values where both methods yield results with comparable accuracy are marked in bold. From left to right, the columns indicate the number of coefficients/basis functions (#coeffs), the number of network weights (#weights), the number of epochs until loss-value stagnation (#epochs), the mean-squared loss value ($\text {MSE}_\text {loss}$), the mean-squared error between the IGANets’ solution and the exact solution interpolated in the spline space ($\text {MSE}_\text {IGANets}$), the number of BiCGStab iterations of the collocation IGA method (#iter), and the mean-squared error between the collocation IGA solution and the exact solution interpolated in the spline space ($\text {MSE}_\text {C-IGA}$)

A direct comparison between IGANets and a regular collocation IGA method is given in Tables 1 and 2 for bi-cubic and bi-quartic tensor-product B-splines, respectively. From left to right, the columns indicate the number of coefficients/basis functions (#coeffs), the number of network weights (#weights), the number of epochs until loss-value stagnation (#epochs), the mean-squared loss value ($\text {MSE}_\text {loss}$), the mean-squared error between the IGANets’ solution and the exact solution interpolated in the spline space ($\text {MSE}_\text {IGANets}$), the number of BiCGStab iterations of the collocation IGA method (#iter), and the mean-squared error between the collocation IGA solution and the exact solution interpolated in the spline space ($\text {MSE}_\text {C-IGA}$). The network is initialized with Xavier-uniform (Glorot) weights and zero bias and optimized using the L-BFGS optimizer with the following configuration: 
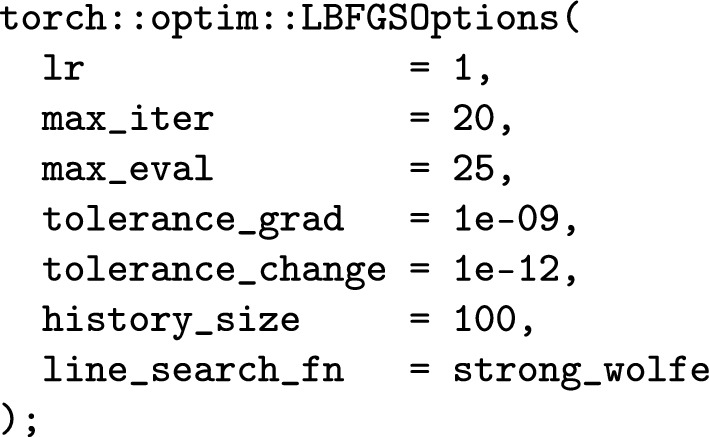


Numerical experiments with alternative optimizers have been performed but demonstrated worse convergence behavior. The training has been stopped after 5000 epochs, which is marked as ’$>5000$’ in Tables 1 and 2. Likewise, the BiCGStab solver was terminated after 10000 iterations, marked as ’$>10000$’ in the aforementioned tables.

It is noteworthy that, in certain cases, already very small networks – one hidden layer with a single neuron – can capture the solution with the same accuracy as the collocation IGA method. In contrast to collocation IGA, IGANets naturally require the use of an optimization algorithm, which not only introduces additional hyperparameters, but also may lead to convergence issues, e.g., apparent in Table 2, where the mean-squared error does not drop below $10^{-12}$. All experiments have been repeated for a larger network with 10 hidden layers and 10 neurons per layer. Next to varying the network configuration, we have performed all experiments with equal weighting of the PDE and boundary loss ($\omega _{\Omega }=1$, $\omega _{\Gamma }=1$) and a much stronger weighting of the boundary loss ($\omega _{\Omega }=1$, $\omega _{\Gamma }=10^3$). While the latter requires more epochs to achieve convergence, it enhances the solution accuracy, especially for the bi-quartic case, cf. Table 2.

## A model problem towards engineering applications

In this section, we demonstrate how IGANets can be used in a typical linear structural mechanics scenario, which represents a common class of PDE problems in engineering. In the following sections, we first describe the underlying governing equations, then present two test cases and conclude with a multi-geometry example demonstrating a potential future engineering workflow.

### Governing and constitutive equations

The equations in this section describe the material behavior under the following assumptions: linear elasticity, plane strain, small strains, isotropic material, homogeneous material, isothermal conditions, no body forces and quasi-static conditions. These simplifications lead to the well-known linear elastic material behavior. In this work, a displacement-based formulation is chosen. Thus, the displacement field ${\textbf{u}}$ serves as the primary unknown, and the corresponding stress distribution is computed during postprocessing via *Hooke’s law*.

The displacement-based formulation and the above-mentioned assumptions combined lead to the static form of the *Navier-Cauchy equations*, consisting of the equilibrium equations, the kinematic relations, and Hooke’s law as the constitutive equation. In the following sections, these individual equations are presented in their strong form.

#### Equilibrium equations

The equilibrium equations for solid mechanics balance inner and outer forces and are (under the condition of no body forces) given by

25 $$\begin{aligned} \nabla \cdot \boldsymbol{\sigma } = {\textbf{0}}, \end{aligned}$$

where $\boldsymbol{\sigma }$ is the Cauchy stress tensor. In two dimensions, with the stress tensor $\boldsymbol{\sigma }$ defined as

26 $$\begin{aligned} \boldsymbol{\sigma } = \begin{bmatrix} \sigma _{xx} & \sigma _{xy} \\ \sigma _{xy} & \sigma _{yy} \end{bmatrix}, \end{aligned}$$

the equilibrium equations can be written in matrix form as

27 $$\begin{aligned} \begin{bmatrix} \frac{\partial \sigma _{xx} }{\partial x} + \frac{\partial \sigma _{xy}}{\partial y} \\ \frac{\partial \sigma _{xy}}{\partial x} + \frac{\partial \sigma _{yy}}{\partial y} \end{bmatrix} = \begin{bmatrix} 0 \\ 0 \\ \end{bmatrix}. \end{aligned}$$

#### Kinematic relations

In linear elasticity under the hypothesis of small strains, the strain tensor is related to the displacement field ${\textbf{u}}$ as

28 $$\begin{aligned} \boldsymbol{\varepsilon } = \frac{1}{2} \left( \nabla {\textbf{u}} + (\nabla {\textbf{u}})^T \right) , \end{aligned}$$

which results in the following matrix expression for the strain tensor $\boldsymbol{\varepsilon }$

29 $$\begin{aligned} \begin{bmatrix} \varepsilon _{xx} & \varepsilon _{xy} \\ \varepsilon _{xy} & \varepsilon _{yy} \end{bmatrix} = \begin{bmatrix} \frac{\partial u_x}{\partial x} & \frac{1}{2} ( \frac{\partial u_x}{\partial y} + \frac{\partial u_y}{\partial x} ) \\ \frac{1}{2} ( \frac{\partial u_x}{\partial y} + \frac{\partial u_y}{\partial x} ) & \frac{\partial u_y}{\partial y} \end{bmatrix}. \end{aligned}$$

#### Constitutive law (Hooke’s law)

For an isotropic, homogeneous, linear elastic material without thermal influence, the stress-strain relationship (Hooke’s law) is

30 $$\begin{aligned} \boldsymbol{\sigma } = \lambda \, \text {tr}(\boldsymbol{\varepsilon }) {\textbf{I}} + 2 \mu \boldsymbol{\varepsilon }, \end{aligned}$$

where $\lambda$ and $\mu$ are the Lamé parameters, and ${\textbf{I}}$ is the identity tensor. The Lamé parameters are material properties and can be evaluated via the material’s Young’s modulus *E* and its Poisson’s ratio $\nu$ in the following relations

31 $$\begin{aligned} \lambda = \frac{E \nu }{(1+\nu )(1-2\nu )} \quad \text {and} \quad \mu = \frac{E}{2(1+\nu )}. \end{aligned}$$

Switching to matrix notation and inserting the values for the strain tensor $\boldsymbol{\varepsilon }$ brings Hooke’s law of (30) into the following form

32 $$\begin{aligned} \begin{bmatrix} \sigma _{xx} & \sigma _{xy} \\ \sigma _{xy} & \sigma _{yy} \end{bmatrix} = \begin{bmatrix} (\lambda + 2 \mu ) \frac{\partial u_x}{\partial x} + \lambda \frac{\partial u_y}{\partial y} & \mu ( \frac{\partial u_x}{\partial y} + \frac{\partial u_y}{\partial x} ) \\ \mu ( \frac{\partial u_x}{\partial y} + \frac{\partial u_y}{\partial x} ) & ( \lambda + 2 \mu ) \frac{\partial u_y}{\partial y} + \lambda \frac{\partial u_x}{\partial x} \end{bmatrix}. \end{aligned}$$

#### Navier-Cauchy equations

The expression previously obtained for the stress tensor $\boldsymbol{\sigma }$ in (32) can now be inserted into the equilibrium condition of (25), resulting in

33 $$\begin{aligned} \begin{bmatrix} (\lambda + 2\mu ) \frac{\partial ^2 u_x}{\partial x^2} + \mu \frac{\partial ^2 u_x}{\partial y^2} + (\lambda + \mu ) \frac{\partial ^2 u_y}{\partial x \partial y} \\ (\lambda + 2\mu ) \frac{\partial ^2 u_y}{\partial y^2} + \mu \frac{\partial ^2 u_y}{\partial x^2} + (\lambda + \mu ) \frac{\partial ^2 u_x}{\partial x \partial y} \end{bmatrix} = \begin{bmatrix} 0 \\ 0 \end{bmatrix}. \end{aligned}$$

Now finally, (33) describes the static form of the *Navier-Cauchy Equations*, which can be, once reorganized, written in compact form as

34 $$\begin{aligned} \mu \nabla ^2 {\textbf{u}} + (\lambda + \mu ) \nabla (\nabla \cdot {\textbf{u}}) = {\textbf{0}}. \end{aligned}$$

### Boundary conditions

The problem is fully defined by specifying boundary conditions (BCs)

35 $$\begin{aligned} {\textbf{u}}&= {\textbf{u}}_D \quad \text {on } \Gamma _D \quad \text {(Dirichlet BC)}, \end{aligned}$$

36 $$\begin{aligned} \boldsymbol{\sigma } \cdot {\textbf{n}}&= {\textbf{t}}_N \quad \text {on } \Gamma _N \quad \text {(Neumann BC)}, \end{aligned}$$

where $\Gamma _D$ and $\Gamma _N$ are the Dirichlet and Neumann boundaries, respectively.

Notice that in collocation methods – in contrast to the finite element method – Neumann boundary conditions are not naturally satisfied and need to be explicitly imposed even in the case of free boundaries [31].

### Single instance example 1: Dirichlet boundary conditions

In order to ensure proper training of IGANets, it is important that the individual loss terms have comparable magnitudes, an issue IGANets share with PINNs. To this end, the loss terms must be appropriately weighted. The purpose of this section is to investigate such weighting strategies.

#### Set-up

**Fig. 2:**
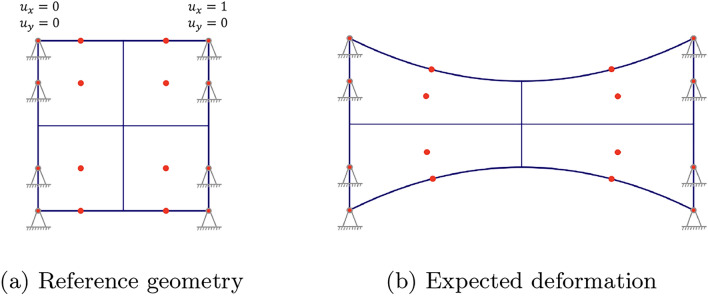
Set-up for numerical example 1 including the reference geometry with Dirichlet BCs in (**a**) and the expected deformation in (**b**)

The computational domain is a unit square with Dirichlet boundary conditions applied on both the left and right edges. On the left edge, the geometry is fixed with zero displacement in both *x*- and *y*-directions. On the right edge, a displacement of 1.0 units is applied in the *x*-direction, while zero displacement is enforced in the *y*-direction. This set-up is illustrated in Fig. 2a. The expected deformation is illustrated in Fig. 2b. The magnitude of the deformation was chosen in such a way that clear displacements can be observed and the approximation errors when comparing different methods become apparent.

#### Determining loss function weights: Dirichlet boundary condition weight as example

**Fig. 3:**
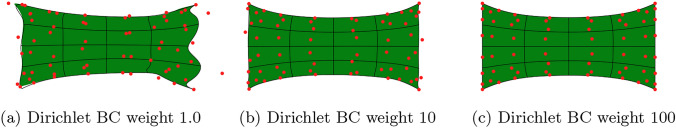
Comparison of different values for the weight of the Dirichlet BC term in the loss function

As discussed in Sect. 2, the weighting of individual contributions to the loss function plays a crucial role in the training process. To illustrate this, we focus on the example of the Dirichlet boundary condition term. The influence of varying this weighting factor is demonstrated in Fig. 3, where three IGANets solutions corresponding to weights of 1, 10, and 100 are compared. The results indicate that a weighting factor of at least 100 is required to achieve a visually satisfactory enforcement of the Dirichlet boundary condition.

**Fig. 4:**
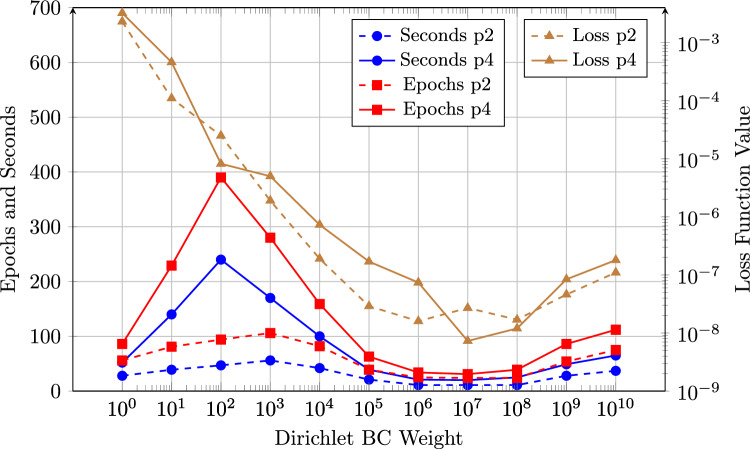
Comparison of the Dirichlet BC weight’s influence on the training behavior of the IGANet in terms of required training time (in seconds) and the number of epochs needed to reach a stationary solution. The comparison was carried out for a spline of degree 2 (p2) and a spline of degree 4 (p4)

To further explore the influence of the weight, a series of simulations was conducted using weighting factors ranging from $10^0$ to $10^{10}$, analyzing their effect on both training performance and solution accuracy (Fig. 4). Note that the IGANet was trained on a standard laptop and the implementation was not optimized. Consequently, the data in Fig. 4 should only be interpreted as a general indication on how the weight influences the training dynamics and final accuracy. The comparison was carried out for two spline types: a degree-2 spline and a degree-4 spline^4^.

For both spline degrees, the training time and the number of epochs are significantly higher for small weights, particularly in the range from $10^1$ to $10^4$. A distinct peak appears at $10^2$ for both training time and epochs, especially for degree 4, indicating a potential instability in the convergence process. However, as the weight increases beyond $10^4$, both the training time and the number of epochs stabilize and reach a minimum around a Dirichlet BC weight of $10^7$.

The loss function values decrease sharply with increasing Dirichlet BC weights and also reach their lowest values around $10^6$ and $10^7$, for degree 2 and 4, respectively. Beyond these BC weights, the residual loss values slightly increase again, which may be attributed to the limitations of double-precision floating-point arithmetic (FP64). Very large BC weights require extremely small loss values to balance the equation, potentially approaching the machine accuracy limit around $10^{-16}$.

When comparing the two spline degrees, degree 4 consistently requires more training time and epochs than degree 2. This is expected, since higher-degree splines have a larger support and therefore more basis functions are nonzero at each collocation point, which increases the cost per training step. Consequently, degree 2 tends to produce lower loss function values in most cases. However, this does not necessarily imply greater accuracy of the resulting solution. For example, reducing the number of control points to just four per direction would probably decrease the residual loss value even further, as a smaller number of collocation points makes it easier for the network to satisfy the governing equations and boundary conditions. Nevertheless, this does not imply that the solution obtained with a 4$\times$4 control point grid would also be more accurate in a physical or an engineering sense.

Overall, the results indicate that the Dirichlet BC weight has a substantial influence on both training efficiency and solution accuracy. In this case, weighting factors below $10^4$ tend to result in unstable training behavior, whereas values in the range of $10^6$ to $10^8$ appear to be optimal. Increasing the weight beyond $10^8$ does not provide further improvements – in fact, it even degrades the solution due to the numerical issues mentioned above. For this reason, a Dirichlet BC weight of $10^7$ is used for the following experiment.

#### Simulation results

**Fig. 5:**
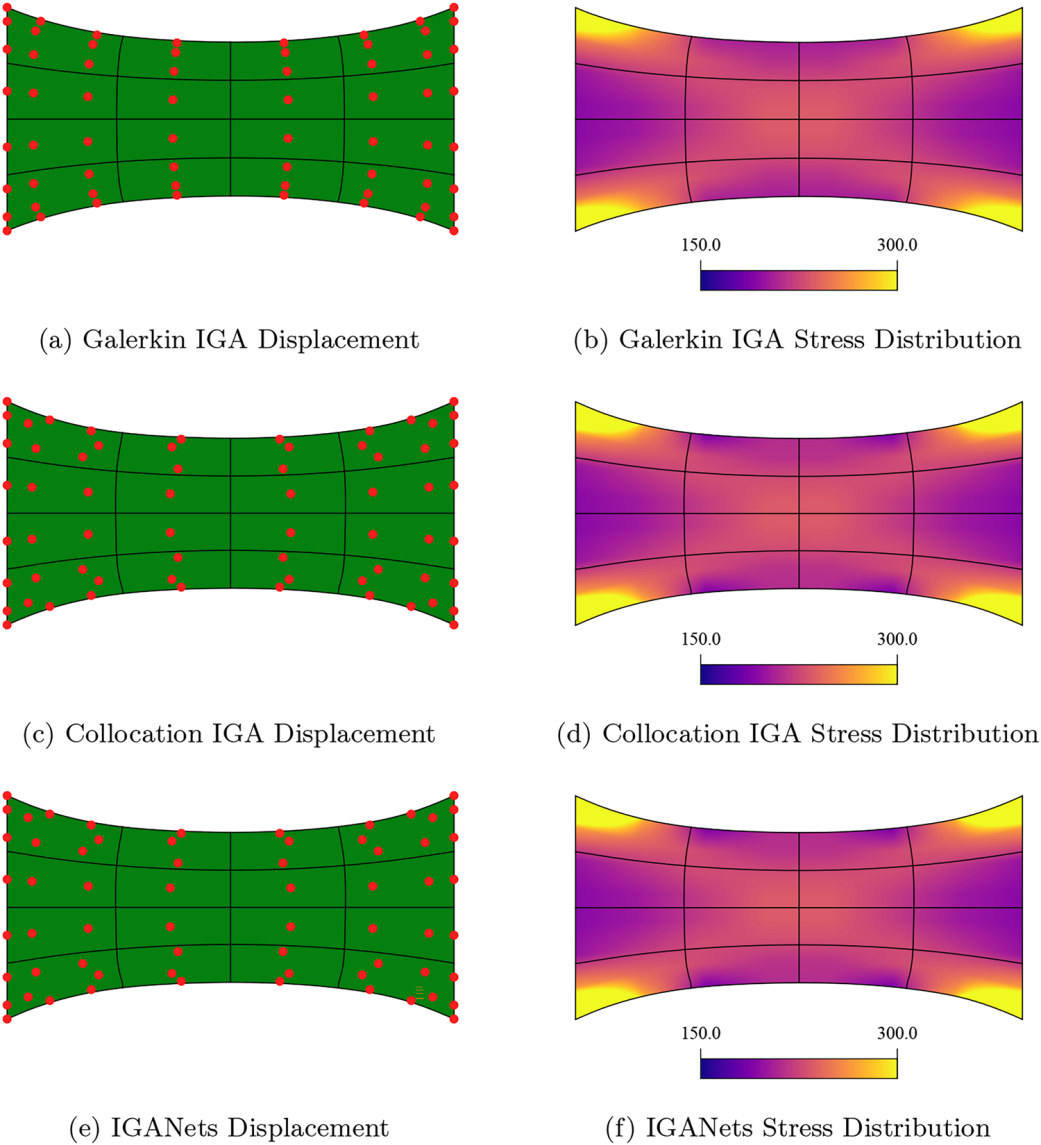
Comparison of different simulation methods in terms of displacement and von Mises stress. Set-up: 8$\times$8 control points and degree 4

In this section, we compare the IGANets results for the test case with a Galerkin-based IGA solution computed in *G+Smo* [32] and a standard collocation IGA solution computed with an in-house *MATLAB* code. In Fig. 5, a first visual comparison of the resulting displacement and stress fields is provided, based on a test set-up of eight control points per direction and a spline degree of 4.

The stresses, visualized in Fig. 5, are limited to a maximum value of 300 in order to account for mathematical singularities occurring at the corners of the domain. These stress peaks grow with increasing mesh resolution or collocation point density and would otherwise dominate the visualization, rendering most of the plot nearly monochrome. At first glance, all three solutions appear visually similar, suggesting that IGANets produce a sufficiently accurate result.

#### Error analysis

**Fig. 6:**
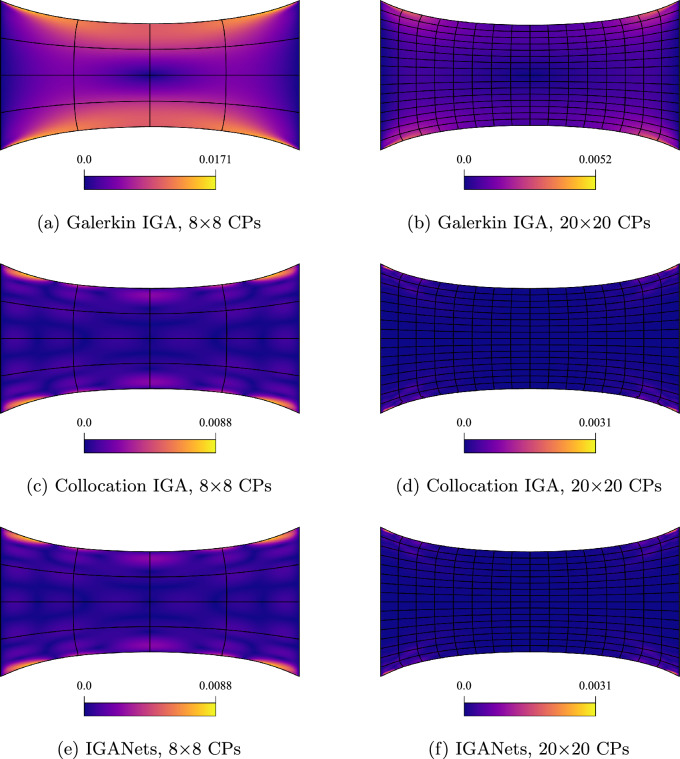
Absolute error distribution with respect to a fine Galerkin IGA simulation for two refinement levels, namely 8$\times$8 and 20$\times$20 control points (CPs)

In order to quantitatively assess the solution quality of IGANets, in Fig. 6 we plot the absolute error for configurations of 8 and 20 control points per direction and spline degree 4 with respect to a very fine Galerkin solution with 64$\times$64 control points. The absolute error is evaluated on a grid of 100$\times$100 evaluation points.

**Fig. 7:**
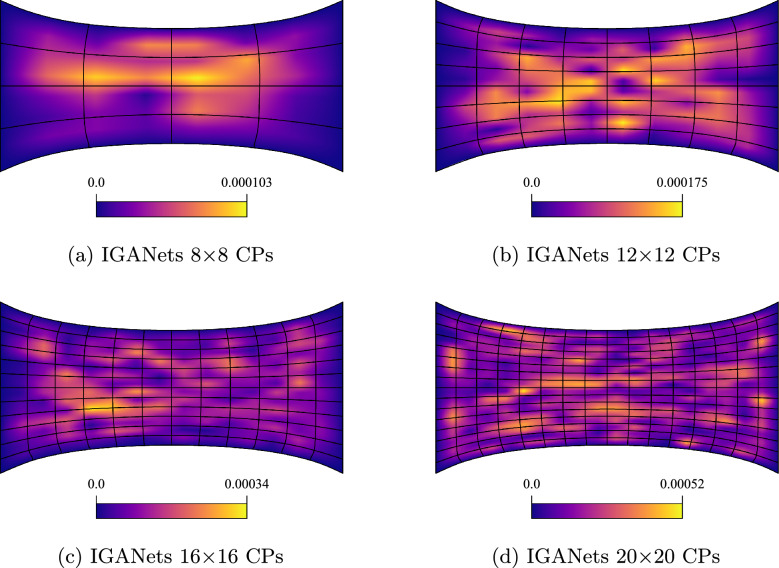
Elasticity error distributions of IGANets for different control point numbers, using a fixed spline degree of $p=4$. Notice that the maximum elasticity error increases with a higher number of control points. While seemingly counter-intuitive, it is in fact plausible, as the network architecture does not change during the experiment and it is easier for the network to satisfy the governing equations at fewer collocation points than across a denser distribution

Within the IGANets framework, the various contributions to the loss function are minimized during training. Unlike the collocation-based reference solution, which enforces the governing equations in strong form and satisfies them exactly at each collocation point, the neural network-based approach of IGANets offers only approximate fulfillment of the physical laws. Consequently, numerical errors can arise due to the inherent approximative nature of the network. Specifically, IGANets minimize the divergence of the stress tensor based on the governing equilibrium equation of linear elasticity, (25), with the aim of reducing this term as close to zero as possible. After training, the residual divergence is evaluated at each collocation point; any nonzero value is interpreted as an error, referred to here as the *Elasticity Error*. The spatial distribution of this error, for various control point configurations, is illustrated in Figure 7. The results indicate that the maximum elasticity error increases with a higher number of control points. This observation appears plausible, as it is easier for the network to satisfy the governing equations at fewer collocation points than across a denser distribution. However, what is particularly noteworthy is the nature of the error distributions. While the solution fields in previous analyses exhibited symmetry in both horizontal and vertical directions, the elasticity error appears non-symmetric across the domain. Despite this, the magnitude of the error remains relatively small, ranging from $1\cdot 10^{-4}$ to $5\cdot 10^{-4}$ and does not significantly affect the overall solution quality. Even in the presence of these residual errors, the geometric behavior of the IGANets solution remains nearly indistinguishable from that of the standard collocation solution.

#### Training behavior

**Fig. 8:**
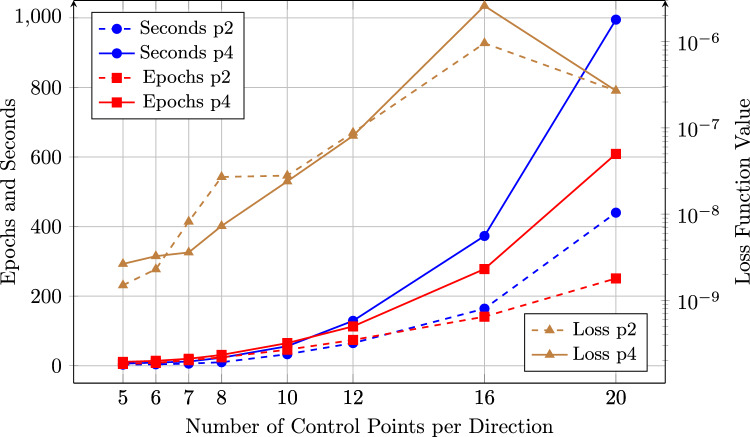
Comparison of the training behavior of IGANets for different control point configurations. The figure illustrates the required training time and number of epochs needed to reach a stationary solution. Additionally, the values of the loss function at these stationary solutions are compared

The IGANet was set up in the following way: The neural network architecture consists of two hidden layers with 25 neurons each, using *Sigmoid* activation functions. A minimum loss threshold of $1\cdot 10^{-8}$ is defined, while the maximum number of epochs is adjusted individually for each simulation run. If the IGANet converges to a stationary solution before reaching the minimum loss, training continues until the specified epoch limit is met. The duration and convergence behavior of the training process depend significantly on the spline degree and the number of control points used. This dependency has been systematically investigated for spline degree 2 and 4 at control point grids ranging from 5$\times$5 CPs to 20$\times$20 CPs. Additionally, the value of the loss function at the stationary solution was recorded in each case. The corresponding results are presented in Figure 8. The figure illustrates that the training time increases exponentially with the number of control points. The same trend is observed for the number of epochs required to reach a stationary solution. For example, considering spline degree 4, the training time increases from 23 seconds for an 8$\times$8 control point grid to 373 seconds for a 16$\times$16 configuration – representing a sixteen-fold increase in time for a four-fold increase in control points. This suggests that the training time scales approximately quadratically with the number of control points in the case of spline degree 4. In addition to training time and epoch count, the values of the loss function at the stationary solution are also shown. For both spline degrees 2 and 4, the plots reveal a general increase in residual loss values with a growing number of control points – up to 16 per direction. Interestingly, for the 20$\times$20 CP case, the loss value decreases again, deviating from the previous trend.

### Single instance example 2: Traction boundary condition

The second experiment within the scope of applying IGANets to computational solid mechanics introduces an external force acting on one of the domain boundaries. As in the previous experiments, the simulation results obtained by IGANets are validated by comparison with the established Galerkin and collocation implementations. In this set-up, the spline degree is fixed to 3 in order to show applicability also to odd degree splines.

A particular focus of this experiment lies on the effect of supervised learning within the IGANets framework. By enabling or disabling the supervised component during training, its impact on solution accuracy and training time can be assessed.

#### Set-up

**Fig. 9:**
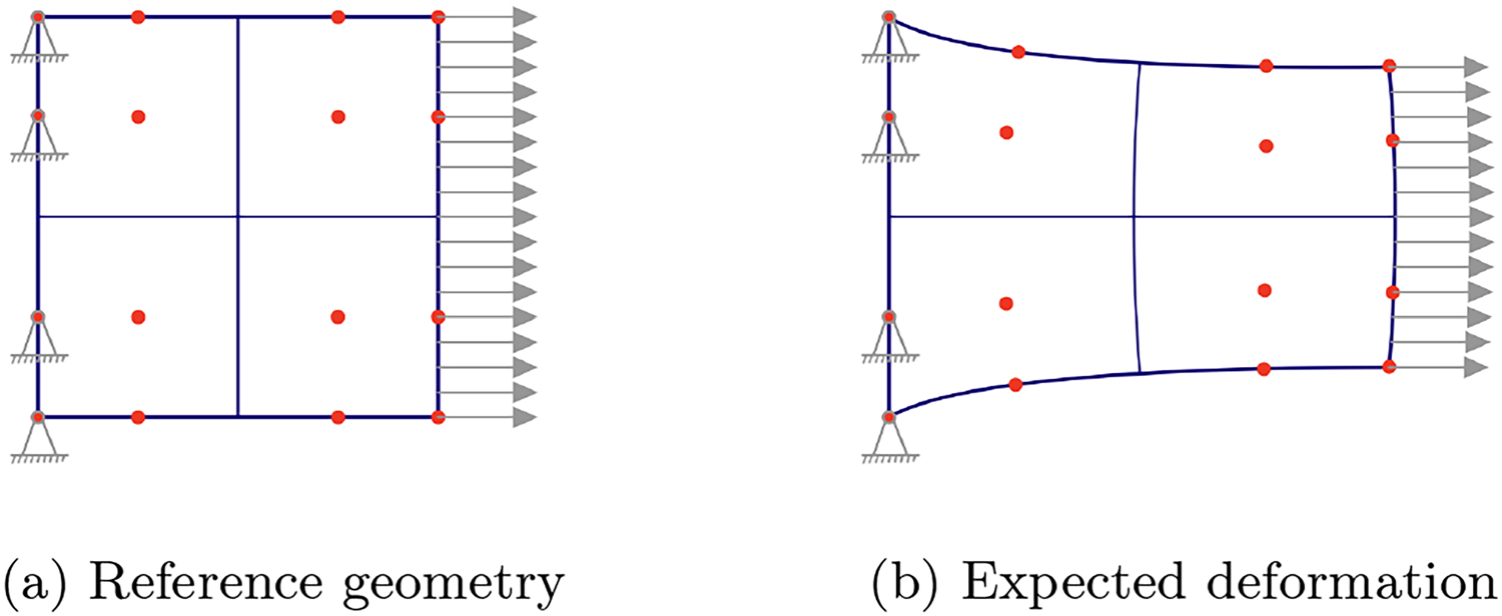
Set-up for numerical example 2 including the reference geometry with Neumann BC in (**a**) and the expected deformation in (**b**)

The reference geometry in this experiment is subject to a zero-displacement Dirichlet BC on the left edge and a traction Neumann BC on the right edge. The traction magnitude is set to 50. As in the previous case, traction-free BCs are applied to the remaining top and bottom edges. The set-up of the experiment is shown in Fig. 9a, and an expected deformation result is illustrated in Fig. 9b. Similarly to the previous test case, the body force ${\textbf{f}}$ is set to zero throughout the domain.

#### Simulation results

**Fig. 10:**
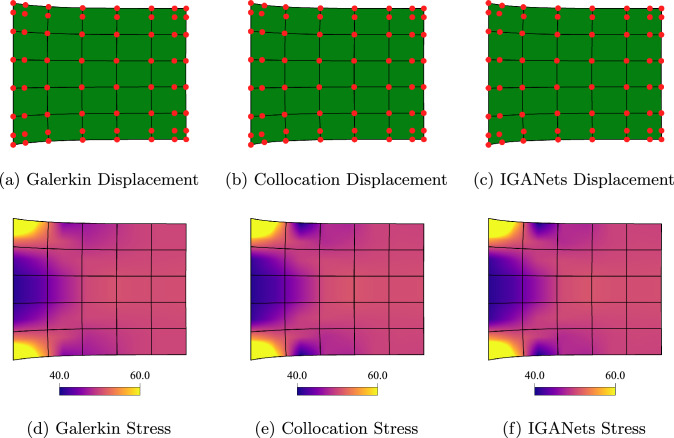
Comparison of the deformation results obtained by a Galerkin-based, a standard collocation, and IGANets simulation (a,b,c), supplemented by their respective stress distribution (d,e,f). Set-up: 8$\times$8 CPs and degree 3

The simulation results of Galerkin IGA, collocation IGA, and IGANets for the test case are presented below. Based on numerical experiments similar to Sect.  4.3.2, a Dirichlet BC weight of $10^8$ has been chosen for the loss function. Figure 10 compares the results obtained by the three simulation methods, including the corresponding stress distributions. Since the traction acts on the right boundary, it is particularly relevant to observe the resulting stress values in this region. Ideally, the stress distribution should reflect this boundary condition and reach values close to 50 near the affected edge.

The stress plots in Fig. 10 (d,e,f) clearly demonstrate that visually, the traction BC on the right edge is fulfilled. Furthermore, the comparison reveals that the results of the standard collocation and IGANets simulation are identical in both displacement and stress distribution for the 8$\times$8 CP configuration.

#### Absolute error distribution

**Fig. 11:**
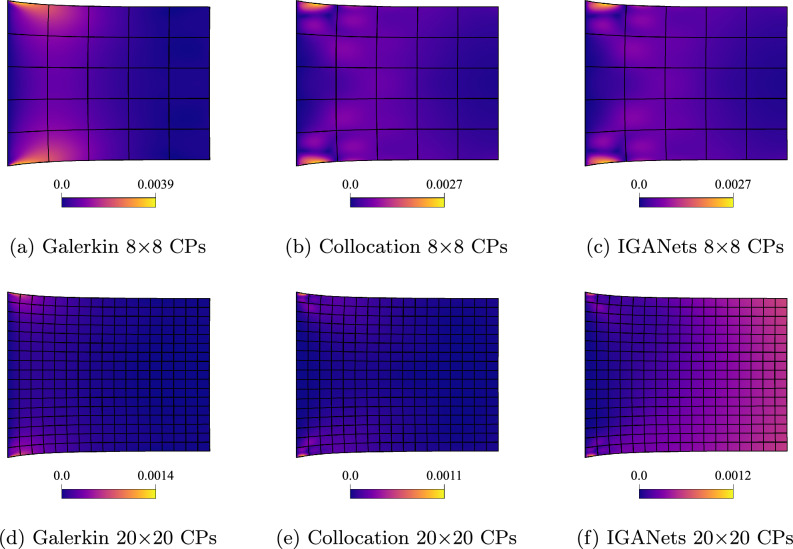
Comparison of the absolute error obtained by Galerkin IGA, collocation IGA, and IGANets against the reference solution for spline degree 3 with 8$\times$8 CPs (**a**, **b**, **c**) and 20$\times$20 CPs (**d**, **e**, **f**)

As in the previous test case, a Galerkin-based reference solution has been computed. For that purpose, a domain with now 100$\times$100 CPs is defined, and the corresponding solution is computed. This high-resolution solution serves as a reference for evaluating the accuracy of the (coarse) Galerkin, collocation, and IGANets simulations. To determine the error, a very dense evaluation grid with $1000 \times 1000$ points is created. At each of these points, the absolute difference between the Galerkin-based reference solution and the corresponding solution of the three methods is calculated. The resulting error distributions are then mapped onto the deformed geometries to visualize where and how the deviations occur. These results are illustrated in Figure 11. The values represent units relative to the domain size. For instance, if the domain is assumed to be 1$\times$1 mm, an absolute error of 0.001 corresponds to a deviation of 1 $\mu$m from the reference solution.

The error plots in Fig. 11 reveal that, for both 8 and 20 CPs per direction, the collocation-based solutions are more accurate than the Galerkin solution computed with G+Smo. Additionally, it becomes evident that the IGANet tends to lose accuracy as the number of control points increases. In the right region of subplot (f), a visible deviation from the other two error distributions with 20 CPs per direction (subplots d and e) can be observed. This trend continues in the following sections, where the IGANet consistently shows slight difficulties in maintaining accuracy for higher control point counts combined with the traction force boundary condition, which might be improved through a more suitable choice of hyperparameters.

#### Simulation results enhanced via supervised learning

In the context of the current traction force experiment, supervised learning has been introduced to support the IGANets training. The goal of supervised learning is to guide the network by providing a known target solution during training. In this case, a standard collocation solution with the same control point number as the current IGANet geometry serves as supervision data. To incorporate this additional information, a supervised loss term is added to the overall loss function. It is computed as the mean squared error (MSE) between the current IGANet control points and those of the collocation-based IGA solution. This term is weighted with a factor larger than 1.0 that controls the influence of the supervised component. A higher weight places greater emphasis on matching the known solution, guiding the network more strongly toward it. In this experiment, the data loss is added alongside the standard components of the traction force experiment: the residuals of the elasticity equations, the traction-free BC, the traction BC and the Dirichlet BC.

#### Training behavior

**Fig. 12:**
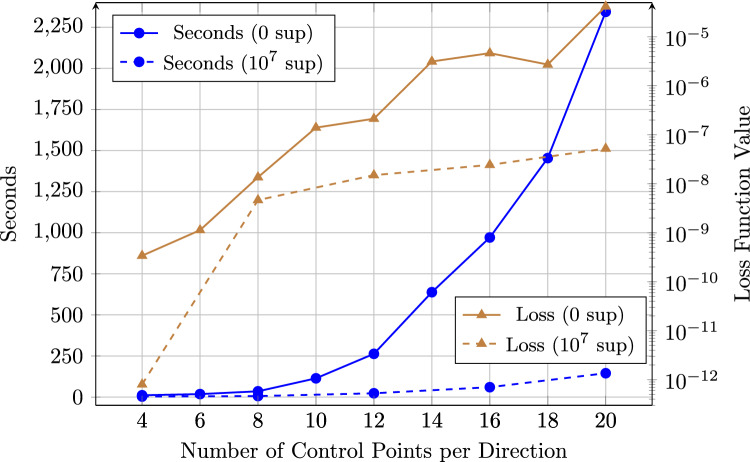
Comparison of the supervised and unsupervised training behavior of IGANets for different control point configurations. The figure illustrates the required training time needed to reach a stationary solution. Additionally, the values of the loss function at these stationary solutions are compared

In this particular experiment, supervised learning proves especially beneficial, as the unsupervised training process is relatively slow. This can be attributed to the additional complexity introduced by the traction force loss, which requires the network to match the applied traction at each collocation point along the right boundary of the domain. To accelerate convergence, an arbitrarily chosen supervised learning weight of $10^7$ is used, in combination with a Dirichlet BC weight of $10^8$. This configuration helps the network prioritize the fulfillment of the Dirichlet and supervised components of the loss. A comparison between the training behavior of supervised and unsupervised runs is shown in Figure 12.

Figure 12 illustrates two key aspects – the exponential increase in training time and the moderate increase of the loss value with growing control point numbers, and the strong effect of supervised learning on reducing both. For instance, while training the IGANet with 20$\times$20 CPs in the unsupervised case takes around 2300 seconds, the supervised version with a weight of $10^7$ reduces this time to only 145 seconds. Despite the typical increase of the residual loss value with more collocation points – caused by the rising complexity of satisfying all conditions – the overall physical solution accuracy still improves with finer discretizations. Remarkably, the supervised version consistently shows lower loss values in all cases, meaning that the governing equations and boundary conditions are better fulfilled compared to the unsupervised set-up.

The use of a relatively high supervised learning weight of $10^7$ is what enables this improvement. If the Dirichlet BC weight is simultaneously reduced (e.g., to 1) and the supervision weight increased further to $10^9$, the training time drops drastically to just 24 seconds for the 20$\times$20 CPs case. Compared to the simulation with a supervision weight of $10^7$, a weight of $10^9$ allows for a further sixfold reduction in training time (24 vs. 145 seconds). One might expect this gain to come at the cost of accuracy. However, the results suggest otherwise as the loss function value also decreases. This is not surprising, as the supervised target – the collocation IGA solution – is already the mathematically best solution the IGANet can approximate. Since both methods are based on the same numerical formulation (collocation), the ideal outcome for the IGANet is to replicate the standard collocation IGA result.

Providing this target has multiple advantages: First, the training time is reduced significantly. Second, the accuracy improves toward the best achievable solution, and third, the cost of obtaining the standard collocation solution is currently negligible, as it can be computed in seconds, even for higher control point resolutions. We conclude that offering a readily available collocation-based reference during training substantially boosts the IGANet’s performance in terms of both computational effort and solution quality.

**Fig. 13:**
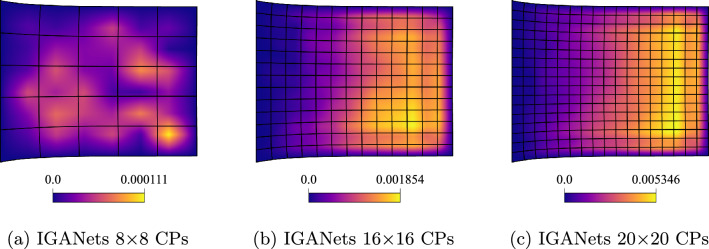
Elasticity error of the **unsupervised** IGANets solution for spline degree 3 at different control point configurations

**Fig. 14:**
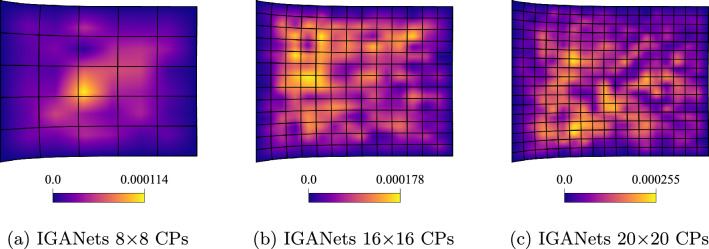
Elasticity error of the **supervised** IGANets solution for spline degree 3 at different control point configurations (supervised learning weight of $10^7$)

To further evaluate the quality of the IGANets solution in both supervised and unsupervised settings, its ability to satisfy the governing equations is analyzed. Recalling the equilibrium condition $\nabla \cdot \boldsymbol{\sigma } = {\textbf{0}}$, any deviation from zero in the divergence of the stress field can be interpreted as an error – previously referred to as the *Elasticity Error*. To visualize this error, the divergence of the stress tensor is computed at each collocation point. This reveals where and to what extent the IGANet struggles to fulfill the equilibrium condition. The results are gathered and compared for both the supervised and unsupervised simulations, providing insight into how supervision affects the model’s ability to satisfy the underlying physics. The corresponding results are shown in Figure 13 and Figure 14.

The unsupervised results in Fig. 13 reveal accuracy issues in the right half of the domain, similar to the behavior observed in the absolute error plots. As the number of control points increases, the elasticity error also grows, since the IGANet struggles to satisfy the governing equations at a higher number of collocation points.

In contrast, the supervised results in Fig. 14 show a similar trend of increasing error with more control points, but at a significantly lower scale. While the unsupervised solution reaches a maximum elasticity error of approximately $5 \cdot 10^{-3}$ for 20$\times$20 CPs, the supervised version shows a much smaller error peak of about $2.5 \cdot 10^{-4}$, making it roughly 20 times more accurate in this case.

These results highlight the strength of supervised learning. Guiding the training towards the standard collocation solution – which represents the best possible outcome for the IGANet – not only reduces training time considerably, but also increases accuracy. Taking these aspects into consideration, the utilization of supervised learning provides significant advantages against the unsupervised IGANet training.

### Multi-instance example: Complex multi-geometry query

To further assess the potential of IGANets for integration into design-oriented workflows, the framework must be evaluated not only on simple benchmark problems but also on more challenging geometries and training scenarios. So far, the numerical examples have been restricted to square domains and single-instance learning, providing a convenient setting to validate the basic methodology. In the following, we therefore consider a more advanced two-dimensional geometry resembling an I-beam. In this setting, particular attention must be paid to the computation of normal vectors required for the traction-free boundary conditions, since the boundary segments are no longer exclusively aligned with straight Cartesian directions. Moreover, while the previous examples kept all network inputs fixed, such that the IGANet effectively acted as solver for a single problem instance, the present study extends this setting to training across multiple geometries and load cases. This allows us to investigate both the approximation accuracy for unseen geometries with respect to an IGA collocation reference solution and the resulting training behavior in a genuine multi-instance setting.

#### Set-up

In this example, we consider a varying geometry, while all instances remain similar to a two-dimensional I-beam. The considered I-beam geometries vary randomly in height and width from $1\times 1$ to $3\times 3$ length units. In this way, a more advanced geometric setting is introduced compared to the previously considered square domains.

The boundary conditions are chosen consistently for all instances. A homogeneous Dirichlet boundary condition is imposed on the bottom edge, traction-free boundary conditions are prescribed on the left and right edges, and a fixed displacement is applied to the top edge. The prescribed displacement is chosen to be comparatively small in order to ensure realistic results within the elastic small-strain regime. More specifically, the displacement of the top edge is varied in both *x*- and *y*-direction within the interval $[-0.1,\,0.1]$.

As in the previous examples, the Dirichlet boundary conditions are imposed weakly during training, using a Dirichlet weight of $10^6$. The collocation points are again chosen as Greville abscissae. However, in contrast to the previous examples, both the geometry and the solution variable are represented using spline spaces of degree 3 with $7 \times 7$ control points.

#### Training method

In this example, the generalization capability of the trained IGANet is investigated. While the previous examples demonstrated that the framework performs very well as an equation solver for a single fixed problem instance, the present setting is designed to evaluate its predictive performance on unseen data.

To this end, the network is trained on datasets consisting of 1, 10, 100, and 1000 training geometries, respectively. These geometries are generated randomly within a prescribed range of geometric variation, yet still living in the same spline space. In addition to the geometry, the boundary conditions are also varied. More specifically, the displacement-based Dirichlet condition on the upper boundary is changed such that the prescribed displacement ranges within $\Delta x, \Delta y \in [-0.1,\,0.1]$.

During training, a set of 100 validation samples is considered. At selected stages of the training process, these validation samples are passed individually through the current IGANet, and the predicted solution coefficients are compared to the corresponding reference solutions. Based on this validation step, training is continued only as long as the network improves its performance on the validation set – in this way, overfitting is avoided.

After training, the final model is evaluated on a set of 25 unseen test samples. The predicted solutions are then compared to the corresponding reference solutions obtained by standard IGA collocation.

#### Simulation results

The considered setting is intended to mimic a simplified engineering workflow. A network is first trained on a set of geometries and boundary conditions within a prescribed range. Afterwards, a new geometry together with new boundary conditions, both lying within this range but not contained in the training or validation data, is provided as input to the trained IGANet. The goal is then to obtain the corresponding deformation result directly from the network without solving a new collocation problem. To assess the quality of this prediction, the resulting deformation is compared to the corresponding standard IGA collocation solution by evaluating the Cartesian error.

More specifically, the following procedure is carried out. First, the network is trained on varying numbers of training samples, including the validation process described above. Next, previously unseen evaluation geometries are generated and assigned boundary conditions within the training range. These geometry and boundary condition coefficients are then provided as inputs to the trained network, yielding a predicted deformation field. Finally, the prediction is compared to the corresponding reference solution obtained by standard IGA collocation.

**Fig. 15:**
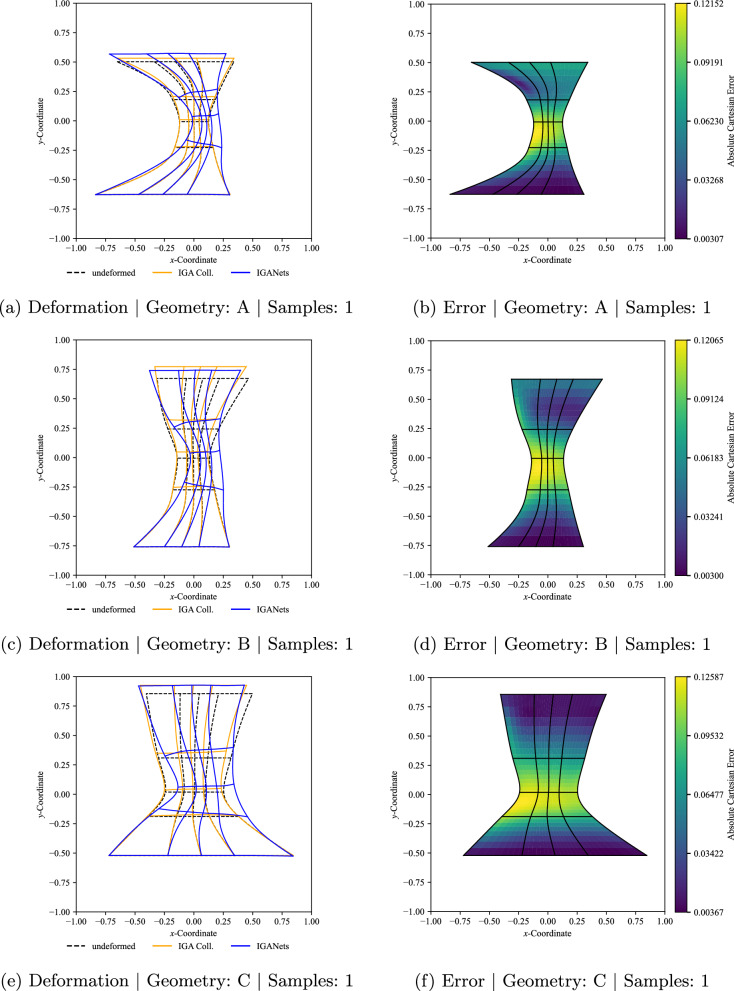
Results of the multi-geometry and multi-boundary condition training for three representative geometries A, B, and C. The IGANets predictions are compared to the corresponding standard IGA collocation solutions. The left-hand side shows the deformed configurations for visual comparison, while the right-hand side presents the Cartesian error. In all cases, the network was trained using only a single training sample

Figure 15 shows the results for three different evaluation geometries, denoted by A, B, and C, when the network has been trained on only a single training sample. It can be seen that the predicted deformations and the collocation reference solutions do not yet coincide perfectly and the network struggles to capture the overall deformation behavior appropriately for the one-training sample case. However, the accuracy improves significantly once the number of training samples is increased.

**Fig. 16:**
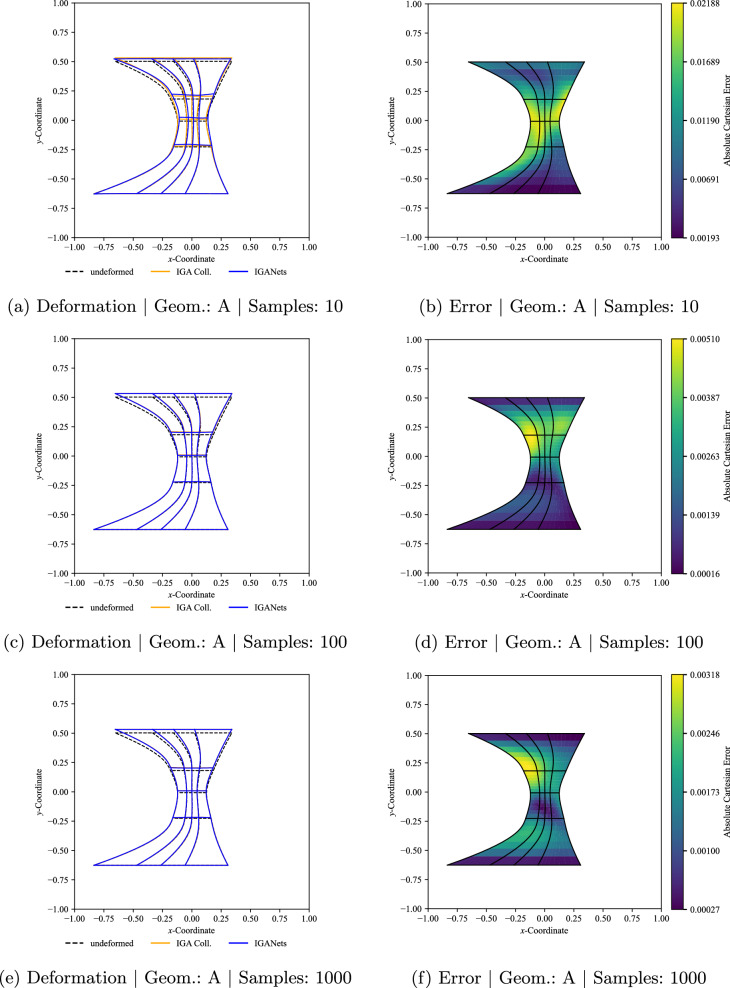
Results of the multi-geometry and multi-boundary condition training for evaluation geometry A. The IGANets predictions are compared to the corresponding standard IGA collocation solutions. The left-hand side shows the deformed configurations for visual comparison, while the right-hand side presents the corresponding Cartesian error. The number of training samples is varied between 10 and 1000

This trend is illustrated further in Fig. 16, now for increasing numbers of training samples. For clarity, not all training set sizes are displayed for every geometry. Instead, the detailed comparison for 10, 100, and 1000 training samples is shown for geometry A only. Overall, the prediction quality remains similar across the considered geometries, indicating that the network performance is not restricted to a specific individual shape. At the same time, a clear improvement in accuracy can be observed with increasing number of training samples. For geometry A, the maximum error is of the order of $10^{-1}$ when training is performed with only one sample, decreases to the order of $10^{-2}$ for 10 training samples, and reaches the order of $10^{-3}$ for both 100 and 1000 samples.

**Fig. 17:**
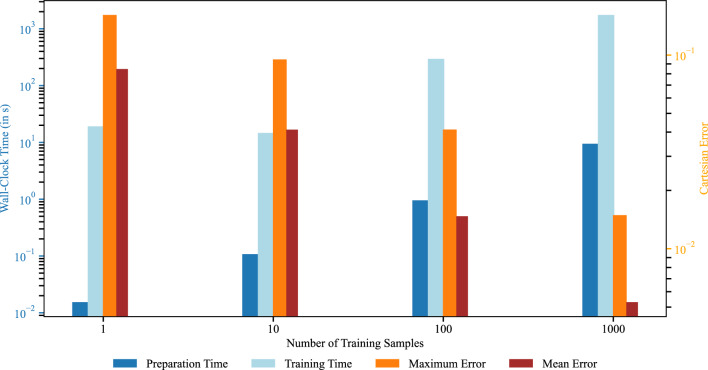
Influence of the number of training samples on computational cost and prediction accuracy in the multi-geometry and multi-boundary condition setting. The left axis shows the wall-clock time for preparation and training, while the right axis reports the maximum and mean Cartesian error over all 25 evaluation samples. With more training samples, the computational cost increases and the prediction error decreases.

To further quantify the effect of the training set size, Fig. 17 summarizes both the computational cost and the prediction accuracy for increasing numbers of training samples. The left axis reports the wall-clock time in seconds, distinguishing between preparation time and training time, while the right axis shows the corresponding Cartesian errors in terms of the maximum and mean error over all 25 evaluation samples. Here, the preparation time denotes the cost of setting up the collocation points and evaluating the required derivatives for each geometry before training. The training time, in contrast, corresponds to the time required to optimize the IGANet on the respective dataset. As expected, both preparation time and training time increase with the number of training samples. At the same time, both error measures decrease consistently, showing that broader coverage of the geometry and boundary-condition space leads to improved predictive accuracy on unseen instances.

A more detailed look at the error measures reveals a clear improvement in generalization performance. The maximum Cartesian error over all evaluation samples decreases with the number of training samples, along with the mean error, following the same trend with lower magnitudes. This indicates that increasing the number of seen geometries and loading conditions substantially improves the robustness and accuracy of the network predictions, although this comes at the expense of higher offline computational cost.

**Fig. 18:**
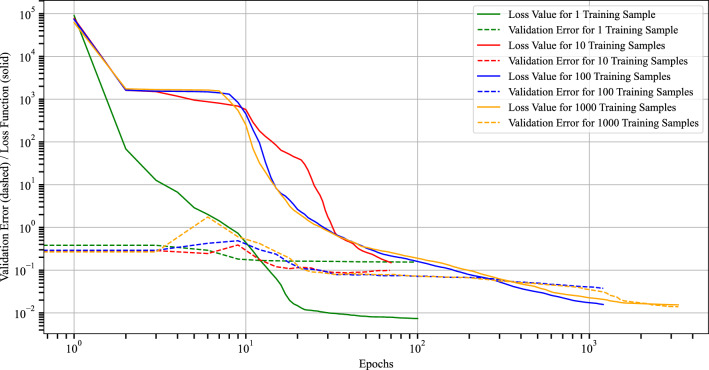
Training loss (solid lines) and validation error (dashed lines) over the epochs for different numbers of training samples in the multi-geometry and multi-boundary condition setting. Smaller training sets lead to faster convergence, while all cases show a substantial loss reduction and a later stabilization of the validation error

The training histories shown in Fig. 18 provide additional insight into this behavior. The solid lines represent the training loss, while the dashed lines denote the validation error, each plotted over the training epochs for training sets ranging from 1 to 1000 samples. The validation error is computed by averaging over all 100 validation samples, where for each sample the corresponding maximum validation error is taken into account. For all cases, the loss decreases significantly over the course of training, indicating that the network successfully fits the underlying problem family. The validation error shows a similar overall trend, although small fluctuations can be observed, particularly during the early stage of training. These variations should be interpreted with care, since the validation error is evaluated only every three epochs, whereas the training loss is recorded at every epoch. Overall, both quantities stabilize at later stages of training, indicating convergence of the optimization process.

A further observation is that smaller training sets lead to substantially shorter training runs in terms of epochs, whereas larger datasets require more epochs before convergence is reached. Together with the results of Fig. 17, this indicates that enlarging the training set primarily improves the predictive quality on unseen geometries while increasing the offline cost of preparing and training the model.

## Conclusions

In this work, we presented IGANets as a prototype digital twin technology that enables rapid evaluation of the physical behavior of new engineering designs. Its ability to approximate PDE solutions with high accuracy makes it a promising tool for both manual use by designers and integration into automated optimization workflows.

IGANets share conceptual ground with PINNs and DeepONets, yet the method distinguishes itself through the explicit use of a spline basis. This choice maintains a direct connection to CAD models, facilitates seamless integration into the design process, and ensures the smoothness required to employ the strong form of the governing PDEs in a collocation setting. Moreover, IGANets build on the theory of IGA collocation, leveraging established results such as the optimal placement of collocation points.

We demonstrated the capabilities of IGANets on two representative PDE problems – the Poisson equation and linear elasticity. Through these examples, we assessed the overall performance of the framework, highlighted the importance of appropriately weighting the individual loss components associated with the PDE and boundary conditions, and showed that the inclusion of even small amounts of labeled data can significantly reduce training time.

Beyond its use as an equation solver for a single fixed problem instance, we further investigated the ability of IGANets to generalize across varying geometries and boundary conditions. For this purpose, a multi-instance linear-elasticity example with I-beam-like geometries was considered. The results showed that IGANets can successfully predict solutions for previously unseen geometries and boundary conditions within the range covered during training. In particular, increasing the number of training samples led to a clear reduction in the prediction error, while naturally increasing the offline effort required for data preparation and training. These findings support the intended use of IGANets in design scenarios in which an offline training phase is followed by rapid online evaluation of new design instances.

Overall, IGANets provide a promising direction for integrating machine learning into engineering analysis and design, combining geometric flexibility with physically informed learning. At the same time, the presented results indicate that its effectiveness in such applications depends strongly on the coverage of the training space in terms of geometry and boundary condition variations. This makes the framework particularly attractive for parametric design studies and digital twin applications, where repeated evaluations within a prescribed design family are required.

## Figures and Tables

**Algorithm 1:**
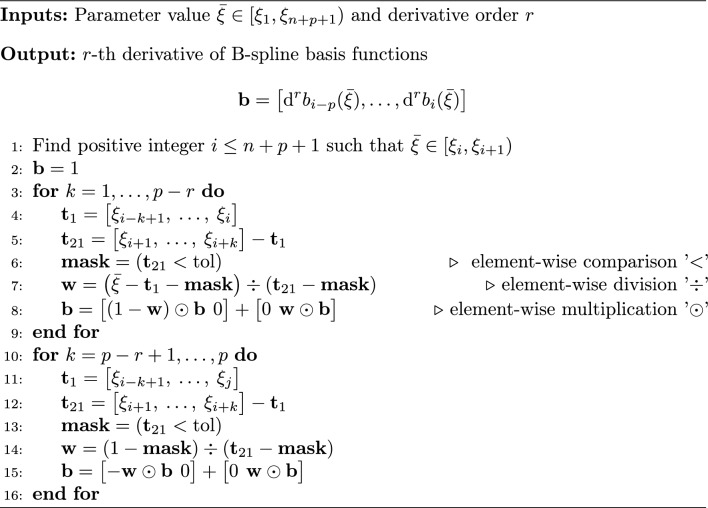
Efficient evaluation of univariate B-spline functions

**Algorithm 2:**
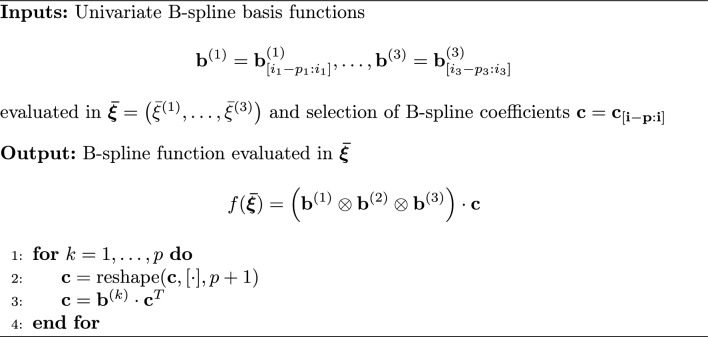
Efficient evaluation of B-spline function (Algorithm 993 [33])

## Data Availability

No datasets were generated or analysed during the current study.

## A Efficient evaluation of B-spline basis functions

For the efficient evaluation of *multi-variate* B-spline basis functions (or their derivatives), Algorithm 1 can be applied to each univariate B-spline basis independently from which the final function value can be computed as follows, e.g.,

37 $$\begin{aligned} f(\boldsymbol{{\bar{\xi }}}) = \left( {\textbf{b}}^{(1)}_{[i_1-p_1:i_1]}({\bar{\xi }}^{(1)}) \otimes \cdots \otimes {\textbf{b}}^{(3)}_{[i_3-p_3:i_3]}({\bar{\xi }}^{(3)}) \right) \cdot {\textbf{c}}_{[{\textbf{i}}-{\textbf{p}};{\textbf{i}}]}. \end{aligned}$$

Here, $[i_\cdot -p_\cdot :i_\cdot ]$ denotes the selection operator that selects the subset of univariate B-spline basis functions ranging from $i-p$ to *i* and $[{\textbf{i}}-{\textbf{p}}:{\textbf{i}}]$ is its multi-dimensional generalization that extracts the corresponding subset of basis coefficients ${\textbf{c}}$.

In a practical implementation, the values of ${\textbf{c}}_{[{\textbf{i}}-{\textbf{p}}:{\textbf{i}}]}$ are typically not stored at contiguous memory positions unless the selection operator makes a deep copy. Often, the data is contiguous in the leading dimension, say, $\xi ^{(1)}$ with offsets of *n* along the second direction, offsets of $n^2$ along the third direction, etc. We therefore suggest using Algorithm 993 [33] for the memory efficient computation of matrix-matrix products with matrices composed of Kronecker products. Dropping the selection operator for ease of readability, (37) can be cast into

38 $$\begin{aligned} \left( {\textbf{b}}^{(1)}\otimes {\textbf{b}}^{(2)} \otimes {\textbf{b}}^{(3)}\right) \cdot {\textbf{c}} = \left( {\textbf{I}}\otimes {\textbf{I}} \otimes {\textbf{b}}^{(3)}\right) \cdot \left( {\textbf{I}}\otimes {\textbf{b}}^{(2)} \otimes {\textbf{I}}\right) \cdot \left( {\textbf{b}}^{(1)}\otimes {\textbf{I}} \otimes {\textbf{I}}\right) \cdot {\textbf{c}}. \end{aligned}$$

This expression can be evaluated in three steps over the number of univariate directions as given in Algorithm 2. The reshape operation in line 2 assumes that matrices are stored in column-major format, that is, after the transposition in line 3, ${\textbf{c}}$ is a matrix with $p+1$ rows. After the successful execution of the algorithm, ${\textbf{c}}$ contains the scalar value of *f* or its derivative in the point $\boldsymbol{{\bar{\xi }}}$.

## Footnotes

1 Mathematically, the operation *flat* corresponds to the standard **vec**torization operation for matrix $\{g_{ij}\}$ and its tensorial counterpart for the tensor $\{{\textbf{x}}_{ij}\}$. Whether row- or column major flattening is performed is irrelevant since the main purpose of flattening is to generate a single input vector for the neural network.

2 The term $3n^2$ comprises the $n^2$ vector-valued coefficients $\{{\textbf{x}}_{ij}\}_{i,j=1}^n$ of the two-dimensional geometry map ${\textbf{x}}_h$ and the scalar coefficients $\{f_{ij}\}_{i,j=1}^n$ of the right-hand side *f*. The term 4*n* results from the scalar coefficients $\{g_i\}_{i=1}^n$ representing the boundary values $g_h$ at the four sides of the boundary $\Gamma$.

3 Mathematically, the *reshape*$(\cdot , n, n)$ operation is the inverse of the *flat* operation as it reinterprets the values of the flat vector as $n\times n$ matrix.

4 Notice that even degree splines are more efficient in collocation than odd degrees.

## Code availability

The IGANets source code is available online at https://github.com/iganets.
